# Machine learning model prediction of 6-month functional outcome in elderly patients with intracerebral hemorrhage

**DOI:** 10.1007/s10143-022-01802-7

**Published:** 2022-05-06

**Authors:** Gianluca Trevisi, Valerio Maria Caccavella, Alba Scerrati, Francesco Signorelli, Giuseppe Giovanni Salamone, Klizia Orsini, Christian Fasciani, Sonia D’Arrigo, Anna Maria Auricchio, Ginevra D’Onofrio, Francesco Salomi, Alessio Albanese, Pasquale De Bonis, Annunziato Mangiola, Carmelo Lucio Sturiale

**Affiliations:** 1 Neurosurgical Unit, Ospedale Spirito Santo, Pescara, Italy; 2 Department of Neurosciences, Imaging and Clinical Sciences, G. D’Annunzio University of Chieti-Pescara, Chieti, Italy; 3 Department of Neurosurgery, Fondazione Policlinico Universitario A. Gemelli IRCSS, Rome, Italy; 4 Department of Neurosurgery, S. Anna University Hospital, Ferrara, Italy; 5 Department of Morphology, Surgery and Experimental Medicine, University of Ferrara, Ferrara, Italy; 6 Department of Anesthesiology, Fondazione Policlinico Universitario A. Gemelli IRCSS, Rome, Italy; 7 Institute of Neurosurgery, Università Cattolica del Sacro Cuore, L.go A. Gemelli 8, 00168 Rome, Italy

**Keywords:** Conventional statistics, Hemorrhagic stroke, Intracerebral hemorrhage, Intracranial hemorrhage, Machine learning, Outcome

## Abstract

Spontaneous intracerebral hemorrhage (ICH) has an increasing incidence and a worse outcome in elderly patients. The ability to predict the functional outcome in these patients can be helpful in supporting treatment decisions and establishing prognostic expectations. We evaluated the performance of a machine learning (ML) model to predict the 6-month functional status in elderly patients with ICH leveraging the predictive value of the clinical characteristics at hospital admission. Data were extracted by a retrospective multicentric database of patients ≥ 70 years of age consecutively admitted for the management of spontaneous ICH between January 1, 2014 and December 31, 2019. Relevant demographic, clinical, and radiological variables were selected by a feature selection algorithm (Boruta) and used to build a ML model. Outcome was determined according to the Glasgow Outcome Scale (GOS) at 6 months from ICH: dead (GOS 1), poor outcome (GOS 2–3: vegetative status/severe disability), and good outcome (GOS 4–5: moderate disability/good recovery). Ten features were selected by Boruta with the following relative importance order in the ML model: Glasgow Coma Scale, Charlson Comorbidity Index, ICH score, ICH volume, pupillary status, brainstem location, age, anticoagulant/antiplatelet agents, intraventricular hemorrhage, and cerebellar location. Random forest prediction model, evaluated on the hold-out test set, achieved an AUC of 0.96 (0.94–0.98), 0.89 (0.86–0.93), and 0.93 (0.90–0.95) for dead, poor, and good outcome classes, respectively, demonstrating high discriminative ability. A random forest classifier was successfully trained and internally validated to stratify elderly patients with spontaneous ICH into prognostic subclasses. The predictive value is enhanced by the ability of ML model to identify synergy among variables.

**Supplementary Information:**

The online version contains supplementary material available at 10.1007/s10143-022-01802-7.

## Introduction

Spontaneous intracerebral hemorrhage (ICH) has an estimated annual incidence of 25/100,000 persons and an overall 12-month mortality of 40–60%, mainly within the first month [2, 21]. The best ICH management remains conservative, while surgery appears controversial and mainly advocated for superficial hemorrhage in younger patients [9, 21, 36].

ICH incidence increases with age, posing a higher assistance burden in countries with aging population [2, 15, 23]. However, besides age, a large number of factors need to be considered for risk stratification, including comorbidities, drugs, ICH volume and site, and neurological status [15].

Functional status rather than mortality should be considered as the correct outcome measurement after ICH, especially in older patients with increasing frailty and reduced long-term expectations. Predicting functional outcome in these patients can be helpful in supporting treatment decisions and prognostic expectations.

Recently, machine learning (ML) has emerged as a powerful tool to develop predictive algorithms from a large amount of data [35].

Here, we built a ML model to predict the 6-month functional status in elderly patients with ICH leveraging the predictive value of the clinical characteristics at hospital admission.

## Methods

### Patient population

We included patients with age ≥ 70 years consecutively admitted at the emergency department of 3 Italian tertiary referral cerebrovascular centers for spontaneous ICH between January 2014 and December 2019.

All the participating hospitals had a 24/7 A&E service and a territory reference stroke unit with an average population above 500,000 persons.

Patients with trauma, ruptured vascular malformations, or hemorrhagic neoplasms were excluded.

Management consisted of surgical evacuation or medical treatment with hyperosmolar solutions, steroids, antiepileptics, head elevation, sedation, and ventilatory and cardiovascular support.

Ethical approval was waived by the local committee due to the retrospective and anonymous nature of the study.

### Data collection

We collected demographical, clinical, and neuroradiological data: patients’ age and gender, comorbidities, current medications, Charlson Comorbidity Index (CCI), and smoking habit; Glasgow Coma Scale (GCS), pupillary size and light reaction, neurological deficits, and seizures at admission; ICH side, location (including lobar, basal ganglia, brainstem, or posterior cranial fossa) and volume measured by the ABC/2 method, and presence of intraventricular hemorrhage; data about surgical or medical treatment; length of hospital stay; and Glasgow Outcome Scale (GOS) at discharge and 6-month follow-up.

### Outcome measures

We identified 3 outcome groups according to 6-month GOS after ICH: dead (D, GOS-1); poor outcome: vegetative status (VS, GOS-2) and severe disability (SD, GOS-3); and good outcome: moderate disability (MD, GOS-4) and good recovery (GR, GOS-5).

We evaluated a ML model feasibility and performance to predict 6-month GOS leveraging the predictive value of patient’s characteristics at admission.

The Transparent Reporting of a multivariable prediction model for Individual Prognosis Or Diagnosis (TRIPOD) statement guidelines were followed to minimize the bias risk during the development phase and correctly validate the predictive ability of our ML models during the testing phase [31].

### Exploratory data analysis

The association between patients’ variables and outcome was investigated with multiple tests (chi-square test, Student’s *t* test, ANOVA, Mann–Whitney *U* test, and Fisher’s exact test). Alfa was set at 0.05, and Holm-Bonferroni correction was applied to shield against type 1 error in the setting of multiple comparisons. All covariates reporting a *p* value < 0.05 at the univariate inferential analysis were further investigated with a multivariable logistic regression model.

### Machine learning model development

**Fig. 1:**
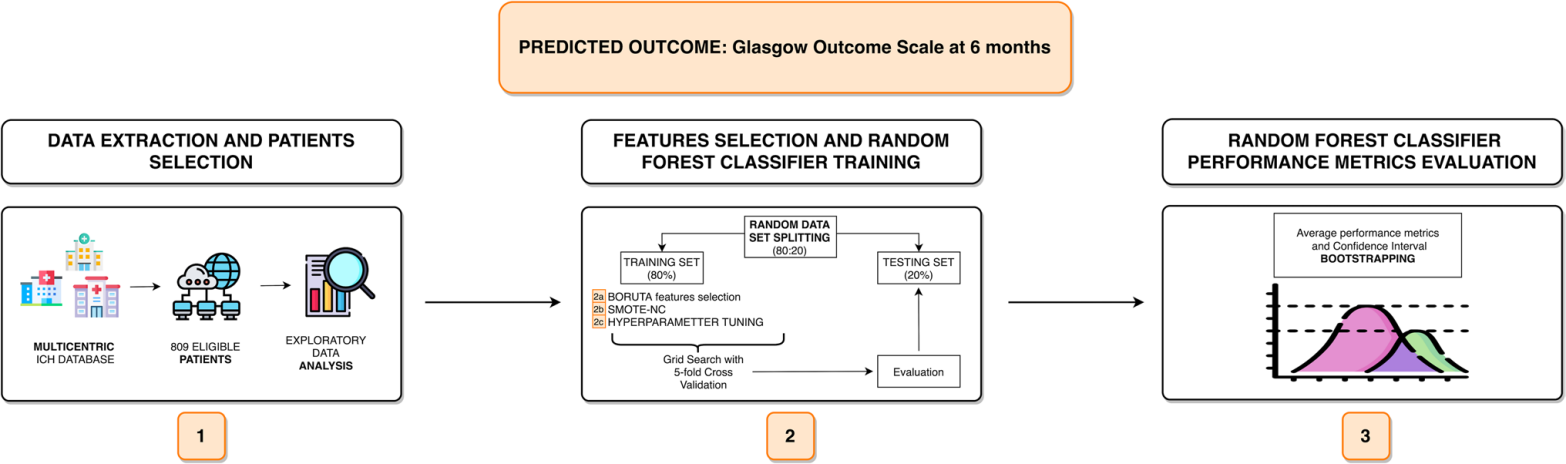
Machine learning workflow. [1] Data extraction and patient selection from a multicentric database. [2] Features selection, hyperparameter tuning, and random forest classifier training. [3] Evaluation of average performance metrics and confidence interval bootstrapping. ICH, intracerebral hemorrhage

The architecture adopted for ML pipeline is outlined in Fig. 1. Considering the characteristics of our dataset, sample size, and outcomes, a random forest (RF) model was deemed the most appropriate ML model [6]. RF is a popular ensemble ML algorithm with easy hyperparameter tuning, lower risk of bias compared to a simple decision tree, elevated generalizability and accuracy, ability to capture non-linear data patterns, and applicability to different data volumes. It works by fitting a number of different decision trees (ensemble): majority “voting”/ “averaging” of all trees’ outcomes are used to classify/predict each patient’s outcome, thus improving predictive accuracy and controlling over-fitting.

Then, the patients’ cohort was randomly split into a training set and a hold-out test set following an 80:20 ratio. No data from the hold-out test set was ever employed during feature selection, synthetic minority over-sampling, and training phase.

#### Feature selection

RF algorithm can achieve reliable performances even when the number of features exceeds that of the dataset instances or when most features are irrelevant to the outcome. Feature selection was performed using Boruta (v.0.3) [26], an all-relevant feature selection algorithm wrapped around RF allowing to identify and retain only the most predictive patient’s variables (input features). Reducing input features shows several advantages: (a) the model’s degrees of freedom reduction decreases the over-fitting and improves the generalizability, and (b) the emphasis of the most important variables enhances the model’s interpretability.

#### Synthetic minority over-sampling technique

Issues deriving from the imbalanced nature of our dataset were explored. When training with imbalanced data, ML algorithms preferentially learnt from the majority than the minority class, thus resulting in limitedly generalizable predictive models. Thus, the original training dataset was balanced using the synthetic minority over-sampling technique-nominal continuous (SMOTE-NC) [8].

#### Random forest training and hyperparameter tuning

The hyperparameter optimization or tuning consists in finding the optimal hyperparameters set for a ML algorithm, which are used to control the learning process. A fivefold cross validation search grid was used on the training set for hyperparameter tuning of RF model. The best performing hyperparameters (e.g., number of estimators, learning rate, max depth) and their spaces tuned via a search grid are reported in the Supplementary Material.

#### Random forest performance metrics evaluation

Finally, the optimized RF model works stratifying hold-out test set patients into prognostic subclasses predicting the 6-month status according to the 3 GOS groups reported above. RF model performance was thoroughly evaluated considering the following metrics:

- Area under the receiving operative characteristics (AUC-ROC)
- Accuracy
- Positive predictive value (PPV) or precision
- Sensitivity or recall
- Specificity
- Negative predictive value (NPV)
- False positive rate (FPR)
- F1 score

A One-vs-the-Rest (OvR) multiclass strategy was employed to extract performance metrics, then the average value and its 95% bootstrap confidence interval were computed.

#### Software

All statistical analyses were performed in Jupyter Notebook, using Python v.3.8.2 whose packages included the following: “Scikit-learn” to develop and train the random forest models, “Numpy” for Excel dataset handling, “imbalanced-learn” to solve class imbalance problem, “Sci-py” to perform univariable statistical association tests, “Statsmodels” to perform multivariable analyses, “Boruta” to perform recursive feature selection, and “LIME” v.0.2.0.1 to interpret the ML model. The entire source code utilized to develop the RF model is available at https://github.com/valerio-mc/ML-in-ICH.

## Results

### Patient population

In this multicenter series, 809 consecutive ICH patients were admitted in a 5-year period (Table 1). Mean age was 79.85 ± 6.35 years, and 389 out of 809 (48.0%) were men. Mean GCS score at admission was 10.43 ± 4.12, while mean ICH score was 1.96 ± 1.5. The mean number of comorbidities was 2.6 ± 1.61 with the most frequents being hypertension (82%), cardiovascular diseases (42%), and diabetes (21%; Supplementary Table 1). The mean number of medications per patient was 3.47 ± 1.98; among them were antihypertensives (72%), antiplatelets (40%), and anticoagulants (21%). Noteworthy, 494 patients (61%) were under anticoagulants and/or antiplatelets. The most frequently ICH topographies were parietal lobes (329 patients, 41%) and cerebellum (99–13%). Only a minority of patients (39/809; 4.8%), generally the youngest (mean 74 years), underwent surgical evacuation.

**Table 1:** Univariate analysis: significant variables

Parameter	Total (*n* = 809)	Dead (*n* = 301)	Poor outcome (*n* = 247)	Good outcome (*n* = 261)	Corrected *p* values
Age	79.85 (± 6.35)	80.89 (± 6.21)	80.85 (± 6.66)	77.72 (± 5.67)	< 0.001*
IVH	215 (27.0%)	129 (42.86%)	56 (22.67%)	30 (11.49%)	< 0.001*
SAH	112 (14.0%)	59 (19.6%)	34 (13.77%)	19 (7.28%)	0.001*
Hematoma volume	35.68 (± 42.14)	61.15 (± 52.38)	26.39 (± 28.21)	15.1 (± 18.47)	< 0.001*
GCS at admission	10.43 (± 4.12)	6.92 (± 3.66)	11.51 (± 3.13)	13.46 (± 1.88)	< 0.001*
ICH score	1.96 (± 1.5)	3.14 (± 1.35)	1.66 (± 1.16)	0.88 (± 0.86)	< 0.001*
Pupillary status at admission					
Isochoric	603 (74.54%)	132 (43.85%)	219 (88.66%)	252 (96.55%)	< 0.001*
Anisocoric	116 (14.34%)	96 (31.89%)	14 (5.67%)	6 (2.3%)	
Mydriatic	45 (5.56%)	39 (12.96%)	5 (2.02%)	1 (0.38%)	
Miotic	45 (5.56%)	34 (11.3%)	9 (3.64%)	2 (0.77%)	
Comorbidities					
Renal insufficiency	51 (6.0%)	17 (5.65%)	9 (3.45%)	25 (10.12%)	0.040*
Neurological	195 (24.0%)	63 (20.93%)	54 (20.69%)	78 (31.58%)	0.026*
Charlson Comorbidity Index	3.36 (± 2.56)	3.34 (± 2.65)	2.79 (± 2.17)	3.99 (± 2.69)	< 0.001*
No. of comorbidities	2.6 (± 1.61)	2.44 (± 1.41)	2.4 (± 1.52)	3.02 (± 1.85)	< 0.001*
Pharmacotherapy					
Antiplatelet	324 (40.0%)	141 (46.84%)	92 (37.25%)	91 (34.87%)	0.046*
Anticoagulant/antiplatelet	494 (61.0%)	216 (71.76%)	143 (57.89%)	135 (51.72%)	< 0.001*
Antacids	193 (24.0%)	98 (32.56%)	53 (21.46%)	42 (16.09%)	< 0.001*
Number of anticoagulants or antiplatelets	0.64 (± 0.56)	0.74 (± 0.55)	0.6 (± 0.54)	0.55 (± 0.57)	0.001*
No. of drugs	3.47 (± 1.98)	3.78 (± 2.01)	3.35 (± 2.03)	3.23 (± 1.85)	0.014*
Topography					
Frontal	226 (28.0%)	121 (40.2%)	62 (25.1%)	43 (16.48%)	< 0.001*
Temporal	223 (28.0%)	113 (37.54%)	67 (27.13%)	43 (16.48%)	< 0.001*
Brainstem	29 (4.0%)	17 (5.65%)	11 (4.45%)	1 (0.38%)	0.016*
Cerebellum	70 (9.0%)	14 (4.65%)	18 (7.29%)	38 (14.56%)	0.001*

Data reported as the number of patients (%) and mean (± SD)

*IVH* intraventricular hemorrhage, *SAH* subarachnoid hemorrhage, *GCS* Glasgow Coma Scale

^*^Significant at *p* ≤ 0.05 after Holm-Bonferroni correction

At 6-month follow-up, 301 patients (37.2%) were dead, 261 (32.3%) had poor outcome (SD-VS), and 247 (30.5%) had good outcome (MD-GR).

### Univariate analysis

At univariate analysis, 20 variables showed a significant association with the 6-month GOS after ICH (Table 1). Mean age, hematoma volume, ICH score, number of drugs, anticoagulants/antiplatelets, comorbidities, and CCI were higher in patients who died at 6 months conversely than mean GCS that was lower. A higher incidence of intraventricular hemorrhage (IVH), subarachnoid hemorrhage (SAH), non-isochoric pupils, and antiplatelet and antacid prescription was reported for patients who died at 6 months. The presence of renal insufficiency and neurological comorbidities was also associated with poor 6-month outcome (GOS 2–3). ICHs involving frontal lobe, temporal lobe, or brainstem were more frequently seen in patients with poorer 6-month outcome.

### Random forest performance metrics evaluation

Features selected for RF classifier via Boruta were age, IVH, hematoma volume, GCS at admission, ICH score, isochoric pupils at admission, anticoagulant/antiplatelet, Charlson Comorbidity Index, and brainstem or cerebellum involvement.

**Fig. 2:**
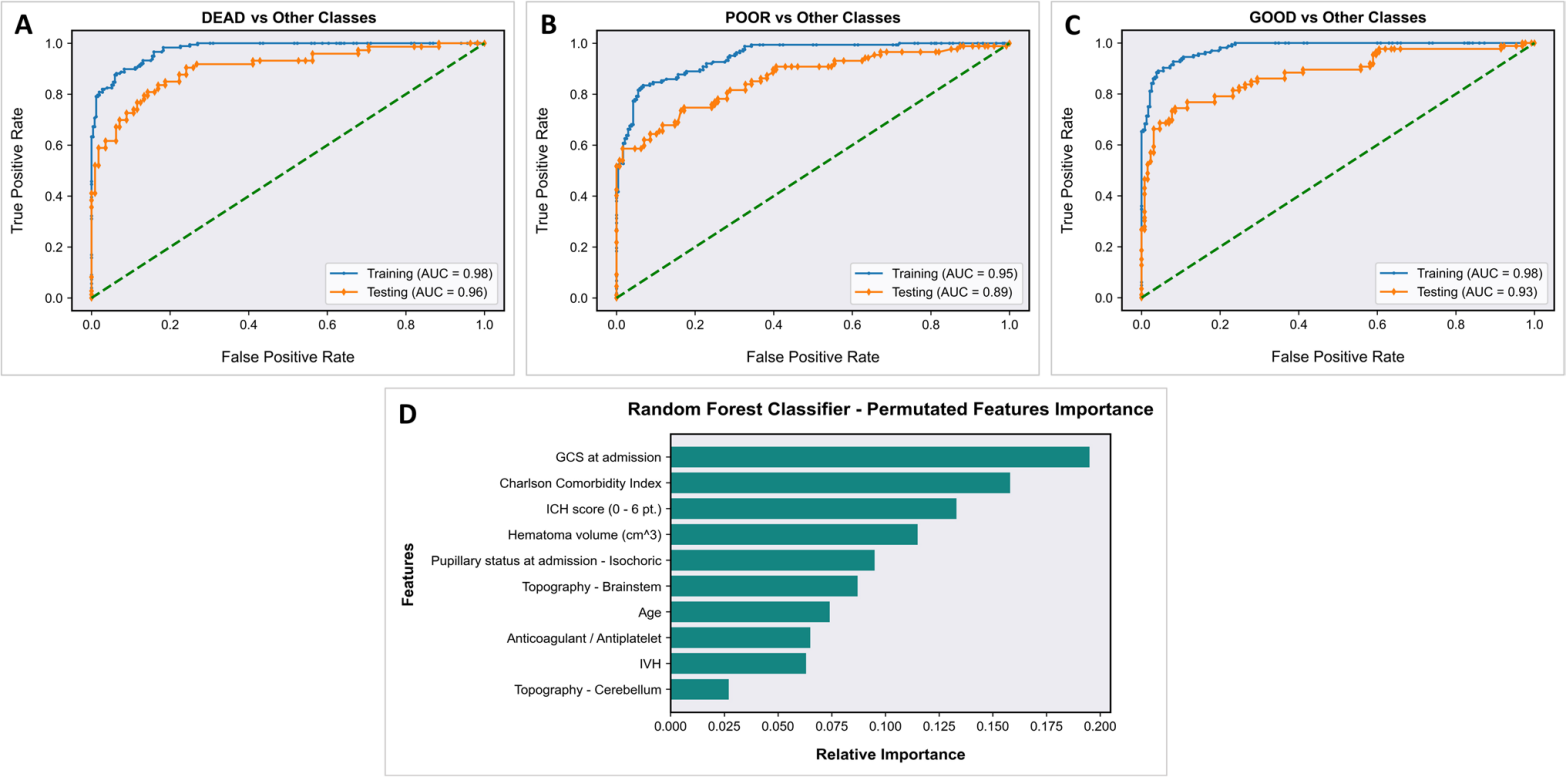
**A**–**C** AUC-ROC curves (on both training and hold-out test set) for each diagnostic outcome class and global confusion matrix. **D** Importance of permutated features for the random forest classifier

RF prediction model, evaluated on the hold-out test set, achieved an AUC of 0.96 (0.94–0.98), 0.89 (0.86–0.93), and 0.93 (0.90–0.95) for dead, poor, and good outcome classes, respectively, demonstrating high discriminative ability (Fig. 2). Moderate to high reliability was reported across all performance metrics for all prognostic outcome classes. Evaluation of the model on the hold-out test set corresponds to internal validation, providing reliable expectation on the model’s performance on new external data (Table 2).

**Table 2:** Random forest prediction model performance metrics

Performance metrics	Dead	Poor outcome	Good outcome
AUC	0.96 (0.94–0.98)	0.89 (0.86–0.93)	0.93 (0.90–0.95)
Accuracy	89.71% (86.42–93.01%)	82.27% (78.60–86.43%)	83.55% (79.82–87.66%)
Precision (PPV)	87.58% (82.69–92.86%)	70.46% (62.69–80.01%)	72.14% (65.59–79.45%)
Recall (sensitivity)	86.92% (80.61–92.86%)	65.04% (55.07–75.36%)	77.52% (68.42–86.84%)
Specificity	91.62% (87.59–95.19%)	89.10% (85.06–93.11%)	86.29% (82.04–91.02%)
F1 score	0.87 (0.83–0.92)	0.68 (0.60–0.76)	0.75 (0.68–0.81)
FPR	8.29% (5.35–14.42%)	11.82% (7.06–16.34%)	14.21% (10.70–19.87%)
NPV	91.75% (88.12–95.24%)	87.05% (83.36–90.21%)	89.52% (86.31–94.26%)

Performance metrics of the random forest prediction model on the hold-out test set were computed adopting a One-vs-Rest (OVR) multiclass strategy. Average value and 95% bootstrap confidence interval are reported

*AUC* area under the curve, *PPV* positive predictive value, *FPR* false positive rate, *NPV* negative predictive value

### Multivariate analysis

At multivariate analysis, several patient variables were significantly associated with 6-month functional status. Results are summarized in Table 3.

**Table 3:** Multivariate logistic regression: significant variables

Outcome	Parameter	Odds ratio	95% CI	*p* value
Death	Cerebellum	0.380	0.143–0.999	0.049
	Age	1.05	1.004–1.097	0.033
	Hematoma volume	1.020	1.008–1.033	0.001
	GCS at admission	0.651	0.574–0.737	< 0.001
	ICH score	1.673	1.187–2.358	0.003
Poor outcome	Brainstem	10.834	1.278–91.851	0.029
	Age	1.057	1.017–1.098	0.005
	Charlson Comorbidity Index	1.211	1.096–1.337	< 0.001
	Hematoma volume	1.012	1.001–1.024	0.036
	GCS at admission	0.760	0.677–0.855	< 0.001

*GCS* Glasgow Coma Scale, *ICH* intracerebral hematoma

Briefly, older age, higher ICH score, and larger ICH volume were associated with the risk of death at 6 months, while cerebellar location and higher GCS at admission were associated with the lower risk of death at 6 months.

Brainstem involvement, older age, higher CCI, and larger hematoma volume were associated with an increased risk of poor 6-month outcome (GOS 2–3). Conversely, higher GCS decreased the risk of poor 6-month outcome.

### Model interpretation

Importance plot of relative features for the RF model is reported in Fig. 2. The parameters with strongest predictive values were as follows: GCS at admission, Charlson Comorbidity Index, ICH score, hematoma volume, pupillary status, and brainstem involvement.

**Fig. 3:**
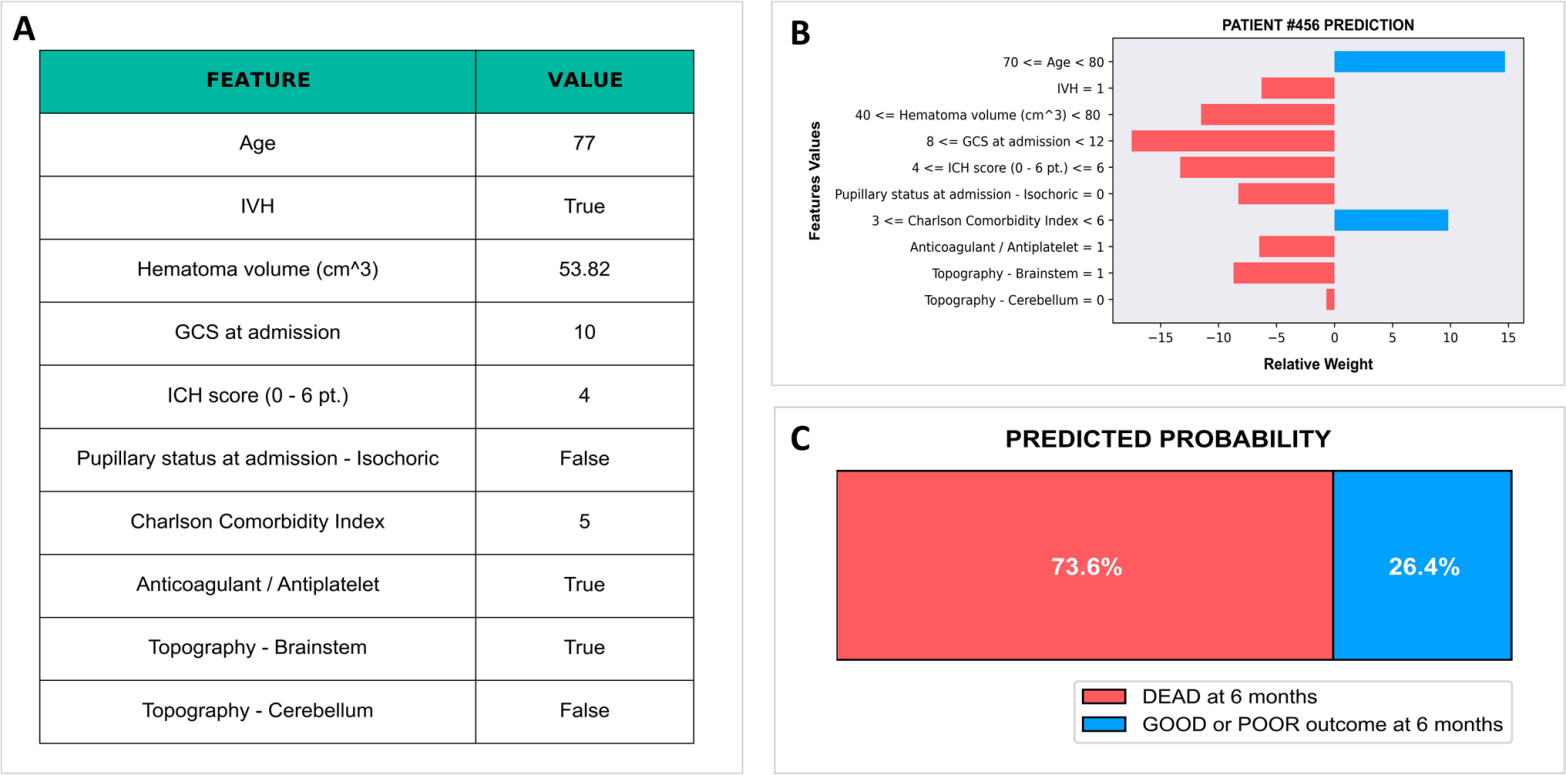
Output of local interpretable model–agnostic explanation (LIME) with random forest classifiers applied to one correctly predicted patient that died within 6 months. The figure reveals the role of various features in influencing the outcome prediction for each patient. **A** Patient’s characteristics. **B** Features contributions on predicted probabilities (red, risk factor; blue, protective factor). **C** Predicted probability of death at 6 months. IVH, intraventricular hemorrhage; GCS, Glasgow Coma Scale; ICH, intracerebral hemorrhage

We further introduced a locally interpretable model-agnostic explanations (LIME) algorithm to quantify the feature contribution and polarity for each patient, thus providing an interpretable relationship between patient’s characteristics and RF model prediction. An example is illustrated in Fig. 3. Understanding the reason behind both correct and incorrect model predictions can increase clinicians’ trust in model behavior and performance.

## Discussion

ICH shows increasing incidence and dismal outcome in elderly population [2, 15, 23]. This is confirmed by our multicenter series showing that about 2/3 of patients died or were in poor functional status after 6 months from ICH presentation. The optimal treatment is still debated for aged patients as, for this population, it is more difficult to determine the prognostic criteria guiding therapeutic choices due to the numerous variables related to their multiple morbidity. For the same reason and the reduced potential of recovery, older patients are also generally poor candidates for surgery [9, 21, 36].

Current treatment algorithms are based on the correction of risk factors to prevent re-bleeding such as a hypocoagulative status and arterial hypertension and on the reduction of secondary damage due to edema and intracranial hypertension [21]. In this scenario, the ability to predict the functional outcome of elderly patients could provide a strong support to the decision-making process regarding management and communication with family.

### Conventional statistics

In this study, we investigated the role of 114 different variables including, among the others, the following: age, drugs, comorbidities, neurological status, as well as some radiological features such as ICH side, location, and volume. Several of these were significantly associated with death or poor 6-month functional outcome at univariate analysis.

Regarding the risk of death, we divided these factors in subject-related (age, number of drugs including antithrombotics, number of comorbidities, and CCI), ICH-related (volume, IVH, SAH), and presenting clinical status–related (GCS, non-isochoric pupils).

Some specific comorbidities such as renal insufficiency and neurological disorders as well as some ICH topographies (frontal, temporal, brainstem) instead appeared related to poor outcome.

At multivariate analysis (Table 3), increasing age, larger hematoma volume, lower GCS at admission, ICH score, and brainstem location were significantly associated with death, poor outcome, or both.

Conversely, cerebellar ICH location was associated with a better clinical outcome at univariate analysis and to a reduced risk of death at multivariate analysis.

### Machine learning model: variable selection and relative importance

A ML model allows to elaborate a prediction algorithm including a number of variables hardly manageable with conventional statistics [22]. In fact, ML also explores non-linear correlations among variables and detects not only significant associations with outcomes, but also the synergy among variables in outcome prediction. Indeed, an electronic health record–based prediction model has been shown to be more accurate in predicting the risk of adverse outcome than traditional models using the a priori selected clinical variables and predictors representing a source of agnostic assessment that is independent of practitioner experience and provide additional assurance to families when considering ongoing intervention [42]. Moreover, the relative weight of single variables may vary among patients due to the interaction and interplay with the others. For example, the use of medications may play different weights among patients’ prognostication according to other features such as age and comorbidities. This allows discovering an unexpected association between variables and outcomes, confirming the role of known variables and emphasizing their combination.

As shown in Fig. 2D, the relative importance of the 10 variables selected by Boruta for our RF model differs with the following order:

1. GCS: presenting GCS shows the higher relative importance in our model. More than 20% of patients have a pre-hospital GCS deterioration (drop of 2 or more GCS points) [30] and another 20% presenting with a GCS score ≥ 13 have an early deterioration after hospital admission [13]. Moreover, elderly patients usually present a worse GCS than younger ones [15]. Baseline GCS and GCS deterioration were found significantly associated with ICH-related mortality and poor functional outcome in several studies [1, 38].
2. CCI: our study confirms previous findings that comorbidities as measured by the CCI independently influence the outcome after ICH [4, 24]. Interestingly, CCI showed a higher relative importance than hematoma volume and patient’s age in our model.
3. ICH score: it is a 6-point score (0-to-5) developed in 2001 to predict the 30-day mortality after ICH [19]. It includes GCS (3–4/5–12/13–15), ICH volume (< 30 ml or > 30 ml), IVH, infratentorial location, and an age threshold of 80 years. This score has also been related to functional outcome in ICH patients [11, 12, 20]. Noticeably, all ICH score variables were automatically selected by our RF model as significant for 6-month outcome prediction. However, some authors observed that the current survival of poor ICH score grades is higher than what prognosticated at the time of score developed 20 years ago [29]. Unlike ICH score using categorical data, the RF model adopts continuous variables as age, ICH volume, and GCS to depict their importance in single patients. The selection of ICH score as feature reinforces the role of other variables.
4. ICH volume: the prognosticating value of ICH volume has been shown as dependent by the topography of the hematoma. Indeed, supratentorial ICH ≥ 30 ml, thalamic ICH ≥ 8 ml, basal ganglia ICH ≥ 18 ml, and cerebellar ICH ≥ 15 ml are cutoff volumes for poor outcome [7, 25, 27]. These differences were not taken into account by the original ICH score.
5. Pupillary status: it is a reliable sign of cerebral herniation and intracranial hypertension. ICH volume and midline shift were correlated with impaired pupillary reactivity [28].
6. Brainstem location: despite often classified as “infratentorial” as well as the cerebellar location, brainstem ICH shows worse clinical presentation and poorer outcome [10, 32].
7. Age: elderly is a risk factor for ICH occurrence and worse outcome [2, 15, 23]. However, it is difficult to distinguish between age itself and associated comorbidities [1]. In our model, the weight of CCI appeared higher than age for outcome prognostication. Indeed, as already shown in several studies on intracranial traumatic and non-traumatic ICH, age is one among, but not the most important risk factor for outcome prediction [3, 37]. In our cohort of elderly patients, the RF model detected an age threshold of 80 years for poor outcome (Fig. 3BB).
8. Anticoagulant/antiplatelet agents: these drugs are associated with an increased risk of ICH occurrence and poor outcome [34, 41]. Despite diverse bleeding odds have been reported with different medications, only antiplatelets showed a significant association with outcome at univariate analysis (Table 1 and Supplementary Table 1), but neither at multivariate statistics nor at RF model. Noticeable, in all participating centers, there was the attitude to pharmacologically reverse the coagulative status, when possible, immediately after the radiological evidence of ICH. However, our ML model automatically selected as significant for outcome prognostication the entire category of anticoagulant/antiplatelet agents.
9. IVH: intraventricular bleeding is more common after thalamic bleeding [27]. The association between IVH and hyperpyrexia and, in turn, their negative influence on prognosis of ICH patients is already known [16, 33].
10. Cerebellar location: cerebellar ICH appeared associated to a better outcome with both conventional statistics and ML mode in agreement with several previous studies [10, 25, 32]. In fact, current American and European guidelines suggest a more aggressive surgical attitude in cases of cerebellar ICH > 3 cm in diameter (about 14 ml) [21, 36].

### Machine learning model: RF model accuracy and clinical implication

We built a robust RF prediction model with high discriminative ability for death (GOS 1), poor outcome (GOS 2–3), and good outcome (GOS 4–5). RF metrics appeared particularly performing in prediction of death (about 90% of accuracy) and both poor and good outcomes (accuracy of 82% and 84%, respectively; Table 2). F1 score, which is a balance between precision and recall, was less reliable for poor outcome detection, due to a relatively high number of false negatives with a recall of 65%. This was balanced by the more satisfactory F1 metrics for the other two outcomes. RF metrics showed that our model has a slightly “optimistic” predictive attitude, with an increasing FPR at improving functional status, suggesting caution in over-emphasizing a good outcome prediction.

### Previous evidences and current novelties

Other authors previously reported their experiences with ML for ICH prognostication.

Wang et al. [39] found that among 39 ML methods, RF was the most accurate for predicting 1-month and 6-month functional outcomes after ICH in a younger cohort of patients.

Similarly, Hall and colleagues [17] demonstrated that hematoma volume, its expansion, GCS score, age, and IVH were the most important variables associated with for outcome prediction at 14-day and 3-month using decision tree and RF models.

Baseline hematoma volume (> 20 ml), IVH, age (> 53 years), and diabetes had the highest percentage of influence weight in a ML model developed by He and colleagues [18] to estimate poor outcome in supratentorial spontaneous ICH treated with conservative treatment. Another ML-based radiomics-clinical model constructed to predict IVH growth after spontaneous ICH found as independent predictors of IVH growth: hypercholesterolemia, baseline Graeb score, time to initial CT, international normalized ratio, and Rad score [43].

Fernandez-Lozano et al. [14] also confirmed that the RF model was the most accurate ML approach, and neurological status during the first 48 h, axillary temperature, early neurological deterioration, leukocyte count, and blood glucose were the most relevant outcome predictors in a large series of patients with ischemic strokes and ICH.

However, all these previous studies focused on a general, unselected, population including younger people and overall agreeing that prognostication in ICH patients is difficult and age is the main functional outcome predictor.

In our study, instead, firstly, we focused the analysis on the elderly population, which is the most often affected by spontaneous ICH and the most problematic for the decision-making process about treatment due to the elevated risk of expected unfunctional outcome.

Our results support the clinicians in defining the expected outcome for ICH patients aged above 70 years.

### Study limitations

Our study has several limitations: first, it has a retrospective nature and limited differences among treatments. Indeed, only a minority of patients underwent ICH evacuation, while the majority had medical treatment, generally based on osmotic agents or hypertonic saline solution at different doses among the participating centers. This made a direct association between treatment and outcome difficult to assess, but assuming that all the cases received the best medical treatment and an eventual evacuation when the clinical status had requested, we believe the results of the present study may reinforce the awareness that some independent variables may play a major role in outcome prediction. In fact, we built a very accurate model able to detect numerous major and minor potential variables, weighting their role not only in dead/alive status prediction, but, above all, also in the 6-month functional status prognostication.

### Perspectives

The input of selected features allows to obtain a predictive probability of the outcome of interest, also allowing to visualize the weight of each variable for single patient prognostication (Fig. 3). This model, available on a freely accessible repository a simple and user-friendly webpage with an interface (3A), could be validated and implemented by external research groups, allowing clinicians to insert details of the 10 selected features of each patient and to calculate the predictive probability for the three main outcomes (dead, poor, and good) at 6 months.

Although the power of prognostication is an undoubtedly important tool helping to assess the severity of illness and provide information to families, the experience and sensitivity of the expert clinicians using these fallible instruments should never be put aside to avoid the generation of a “self-fulfilling prophecy” attitude discouraging from the effort to maximize the treatment opportunities for patients, which is the aim of this study [5, 29, 40].

## Conclusions

A RF classifier was successfully trained and internally validated to stratify elderly patients with spontaneous ICH into prognostic subclasses based on GOS. The predictive value is enhanced by the ability of the ML model to identify factors not relevant if analyzed singularly, but becoming significant when combined each other in a real-life setting.

## Supplementary Information

Below is the link to the electronic supplementary material.

Supplementary file1 (DOCX 32 KB)

Supplementary file2 (DOCX 19 KB)

## Data Availability

Available upon reasonable request.
