# Supplementary material for: Machine learning model prediction of 6-month functional outcome in elderly patients with intracerebral hemorrhage
Source: Neurosurg Rev. 2022 May 6;45(4):2857–67. doi: 10.1007/s10143-022-01802-7 (PMC9349060; doi:10.1007/s10143-022-01802-7)
Supplement: Supplementary file 1 — Supplementary file1 (DOCX 32 KB) [file 10143_2022_1802_MOESM1_ESM.docx]

**Supplementary Table 1.** Baseline parameters of the 809 included patients.

| **Parameter** | | **Total**  **(n=809)** | **Dead**  **(n=301)** | **Poor outcome**  **(n=247)** | **Good outcome**  **(n=261)** | **Corrected p-values** |
| --- | --- | --- | --- | --- | --- | --- |
| Gender (Male) | | 389 (48.0%) | 155 (51.5%) | 100 (40.49%) | 134 (51.34%) | 0.083 |
| Age | | 79.85 (± 6.35) | 80.89 (± 6.21) | 80.85 (± 6.66) | 77.72 (± 5.67) | **< 0.001*** |
| IVH | | 215 (27.0%) | 129 (42.86%) | 56 (22.67%) | 30 (11.49%) | **< 0.001*** |
| SAH | | 112 (14.0%) | 59 (19.6%) | 34 (13.77%) | 19 (7.28%) | **0.001*** |
| Hematoma volume (cm^3^) | | 35.68 (± 42.14) | 61.15 (± 52.38) | 26.39 (± 28.21) | 15.1 (± 18.47) | **< 0.001*** |
| GCS at admission | | 10.43 (± 4.12) | 6.92 (± 3.66) | 11.51 (± 3.13) | 13.46 (± 1.88) | **< 0.001*** |
| ICH score (0 - 5 pt.) | | 1.96 (± 1.5) | 3.14 (± 1.35) | 1.66 (± 1.16) | 0.88 (± 0.86) | **< 0.001*** |
| Pupillary status at admission | Isochoric | 603 (74.54%) | 132 (43.85%) | 252 (96.55%) | 219 (88.66%) | **< 0.001*** |
|  | Anisocoric | 116 (14.34%) | 96 (31.89%) | 6 (2.3%) | 14 (5.67%) |  |
|  | Mydriatic | 45 (5.56%) | 39 (12.96%) | 1 (0.38%) | 5 (2.02%) |  |
|  | Miotic | 45 (5.56%) | 34 (11.3%) | 2 (0.77%) | 9 (3.64%) |  |
| **Comorbidities** | | | | | | |
| Hypertension | | 660 (82.0%) | 248 (82.39%) | 197 (79.76%) | 215 (82.38%) | 0.768 |
| Diabetes | | 169 (21.0%) | 58 (19.27%) | 54 (21.86%) | 57 (21.84%) | 0.769 |
| Allergies | | 2 (0.0%) | 2 (0.66%) | 0 (0.0%) | 0 (0.0%) | 0.428 |
| Respiratory | | 75 (9.0%) | 30 (9.97%) | 23 (9.31%) | 22 (8.43%) | 0.888 |
| Cardiovascular | | 336 (42.0%) | 131 (43.52%) | 112 (45.34%) | 93 (35.63%) | 0.192 |
| Cutaneous | | 3 (0.0%) | 1 (0.33%) | 1 (0.4%) | 1 (0.38%) | 1.00 |
| Hematological | | 42 (5.0%) | 12 (3.99%) | 15 (6.07%) | 15 (5.75%) | 0.598 |
| Endocrinological | | 90 (11.0%) | 25 (8.31%) | 33 (13.36%) | 32 (12.26%) | 0.343 |
| Gastroenteric | | 102 (13.0%) | 29 (9.63%) | 37 (14.98%) | 36 (13.79%) | 0.343 |
| Genito-urinary | | 62 (8.0%) | 18 (5.98%) | 21 (8.5%) | 23 (8.81%) | 0.527 |
| Ictus / TIA | | 119 (15.0%) | 49 (16.28%) | 43 (17.41%) | 27 (10.34%) | 0.179 |
| Infectious | | 26 (3.0%) | 8 (2.66%) | 14 (5.67%) | 4 (1.53%) | 0.112 |
| Dyslipidemia | | 73 (9.0%) | 18 (5.98%) | 29 (11.74%) | 26 (9.96%) | 0.181 |
| Renal Insufficiency | | 51 (6.0%) | 17 (5.65%) | 25 (10.12%) | 9 (3.45%) | **0.040*** |
| Neurological | | 195 (24.0%) | 63 (20.93%) | 78 (31.58%) | 54 (20.69%) | **0.026*** |
| Cerebrovascular | | 59 (7.0%) | 22 (7.31%) | 18 (7.29%) | 19 (7.28%) | 1.000 |
| Overweight or Obesity | | 14 (2.0%) | 7 (2.33%) | 5 (2.02%) | 2 (0.77%) | 0.492 |
| Ocular | | 55 (7.0%) | 17 (5.65%) | 21 (8.5%) | 17 (6.51%) | 0.552 |
| Oncological | | 133 (16.0%) | 36 (11.96%) | 50 (20.24%) | 47 (18.01%) | 0.112 |
| Osteoarticular | | 64 (8.0%) | 19 (6.31%) | 25 (10.12%) | 20 (7.66%) | 0.492 |
| ENT | | 14 (2.0%) | 6 (1.99%) | 4 (1.62%) | 4 (1.53%) | 0.94 |
| Rheumatological | | 63 (8.0%) | 19 (6.31%) | 18 (7.29%) | 26 (9.96%) | 0.492 |
| DVT or pulmonary embolism | | 14 (2.0%) | 6 (1.99%) | 6 (2.43%) | 2 (0.77%) | 0.492 |
| Bed ridden | | 1 (0.0%) | 0 (0.0%) | 1 (0.4%) | 0 (0.0%) | 0.492 |
| Alcohol | | 2 (0.0%) | 1 (0.33%) | 0 (0.0%) | 1 (0.38%) | 0.735 |
| Smoke | | 156 (19.0%) | 66 (21.93%) | 43 (17.41%) | 47 (18.01%) | 0.492 |
| Charlson Comorbidity Index | | 3.36 (± 2.56) | 3.34 (± 2.65) | 3.99 (± 2.69) | 2.79 (± 2.17) | **< 0.001*** |
| N° Comorbidities | | 2.6 (± 1.61) | 2.44 (± 1.41) | 3.02 (± 1.85) | 2.4 (± 1.52) | **< 0.001*** |
| **Pharmacotherapy** | | | | | | |
| Anticoagulant | | 170 (21.0%) | 75 (24.92%) | 51 (20.65%) | 44 (16.86%) | 0.201 |
| Antiplatelet | | 324 (40.0%) | 141 (46.84%) | 92 (37.25%) | 91 (34.87%) | **0.046*** |
| Biliary salts | | 12 (1.0%) | 5 (1.66%) | 4 (1.62%) | 3 (1.15%) | 0.909 |
| Analgesic opioids | | 7 (1.0%) | 2 (0.66%) | 3 (1.21%) | 2 (0.77%) | 0.858 |
| Anxiolytic | | 46 (6.0%) | 19 (6.31%) | 9 (3.64%) | 18 (6.9%) | 0.492 |
| Antianginal | | 20 (2.0%) | 9 (2.99%) | 3 (1.21%) | 8 (3.07%) | 0.492 |
| Anticoagulant/Antiplatelet | | 494 (61.0%) | 216 (71.76%) | 143 (57.89%) | 135 (51.72%) | **< 0.001*** |
| Antihistamines | | 2 (0.0%) | 2 (0.66%) | 0 (0.0%) | 0 (0.0%) | 0.428 |
| Antiandrogens | | 4 (0.0%) | 1 (0.33%) | 0 (0.0%) | 3 (1.15%) | 0.389 |
| Antianemia | | 31 (4.0%) | 14 (4.65%) | 11 (4.45%) | 6 (2.3%) | 0.492 |
| Antiarrhythmic | | 62 (8.0%) | 32 (10.63%) | 15 (6.07%) | 15 (5.75%) | 0.179 |
| Bronchodilator | | 22 (3.0%) | 12 (3.99%) | 5 (2.02%) | 5 (1.92%) | 0.492 |
| Antibiotics | | 23 (3.0%) | 14 (4.65%) | 3 (1.21%) | 6 (2.3%) | 0.171 |
| Antifungal | | 1 (0.0%) | 1 (0.33%) | 0 (0.0%) | 0 (0.0%) | 0.561 |
| Anticholinergic | | 1 (0.0%) | 1 (0.33%) | 0 (0.0%) | 0 (0.0%) | 0.561 |
| Antidementia | | 39 (5.0%) | 16 (5.32%) | 14 (5.67%) | 9 (3.45%) | 0.567 |
| Antidepressant | | 118 (15.0%) | 52 (17.28%) | 28 (11.34%) | 38 (14.56%) | 0.364 |
| Antihemorrhagic | | 1 (0.0%) | 0 (0.0%) | 0 (0.0%) | 1 (0.38%) | 0.492 |
| Antiglaucoma | | 20 (2.0%) | 10 (3.32%) | 3 (1.21%) | 7 (2.68%) | 0.492 |
| Antigout | | 58 (7.0%) | 22 (7.31%) | 22 (8.91%) | 14 (5.36%) | 0.492 |
| Anti-BPH | | 61 (8.0%) | 31 (10.3%) | 13 (5.26%) | 17 (6.51%) | 0.201 |
| Antihypertensive | | 586 (72.0%) | 218 (72.43%) | 177 (71.66%) | 191 (73.18%) | 0.956 |
| Anti-hyperkalemia | | 5 (1.0%) | 4 (1.33%) | 0 (0.0%) | 1 (0.38%) | 0.328 |
| Antimalarial | | 1 (0.0%) | 0 (0.0%) | 1 (0.4%) | 0 (0.0%) | 0.492 |
| Chemotherapy | | 12 (1.0%) | 2 (0.66%) | 4 (1.62%) | 6 (2.3%) | 0.492 |
| Antiepileptic | | 76 (9.0%) | 26 (8.64%) | 29 (11.74%) | 21 (8.05%) | 0.492 |
| Antiparkinsonian | | 35 (4.0%) | 8 (2.66%) | 16 (6.48%) | 11 (4.21%) | 0.270 |
| Antipsychotic | | 48 (6.0%) | 26 (8.64%) | 13 (5.26%) | 9 (3.45%) | 0.133 |
| Urinary antispasmodics | | 3 (0.0%) | 0 (0.0%) | 3 (1.21%) | 0 (0.0%) | 0.139 |
| Antithyroid | | 7 (1.0%) | 4 (1.33%) | 2 (0.81%) | 1 (0.38%) | 0.596 |
| Anti-skin ulcers | | 1 (0.0%) | 0 (0.0%) | 1 (0.4%) | 0 (0.0%) | 0.492 |
| Antivirals | | 2 (0.0%) | 1 (0.33%) | 1 (0.4%) | 0 (0.0%) | 0.711 |
| Antidotes | | 3 (0.0%) | 0 (0.0%) | 0 (0.0%) | 3 (1.15%) | 0.168 |
| Calcium antagonists | | 4 (0.0%) | 3 (1.0%) | 0 (0.0%) | 1 (0.38%) | 0.492 |
| Endothelial protector | | 6 (1.0%) | 4 (1.33%) | 1 (0.4%) | 1 (0.38%) | 0.492 |
| Corticosteroids | | 21 (3.0%) | 10 (3.32%) | 9 (3.64%) | 2 (0.77%) | 0.232 |
| Decongestant | | 0.0 (0.0%) | 0.0 (0.0%) | 0.0 (0.0%) | 0.0 (0.0%) | 1 |
| Diuretics | | 171 (21.0%) | 66 (21.93%) | 58 (23.48%) | 47 (18.01%) | 0.492 |
| Pancreatic enzymes | | 1 (0.0%) | 0 (0.0%) | 0 (0.0%) | 1 (0.38%) | 0.492 |
| NSAID | | 19 (2.0%) | 7 (2.33%) | 7 (2.83%) | 5 (1.92%) | 0.864 |
| Laxatives | | 11 (1.0%) | 6 (1.99%) | 3 (1.21%) | 2 (0.77%) | 0.567 |
| Enzyme inhibitor | | 2 (0.0%) | 0 (0.0%) | 1 (0.4%) | 1 (0.38%) | 0.671 |
| Antacids | | 193 (24.0%) | 98 (32.56%) | 53 (21.46%) | 42 (16.09%) | **< 0.001*** |
| Immunosuppressors | | 1 (0.0%) | 0 (0.0%) | 1 (0.4%) | 0 (0.0%) | 0.492 |
| Insulin | | 49 (6.0%) | 19 (6.31%) | 19 (7.69%) | 11 (4.21%) | 0.492 |
| Supplements | | 28 (3.0%) | 11 (3.65%) | 5 (2.02%) | 12 (4.6%) | 0.492 |
| Hypnotic | | 20 (2.0%) | 5 (1.66%) | 6 (2.43%) | 9 (3.45%) | 0.543 |
| Oral antiglycemics | | 116 (14.0%) | 53 (17.61%) | 28 (11.34%) | 35 (13.41%) | 0.286 |
| Lipid-lowering | | 185 (23.0%) | 72 (23.92%) | 55 (22.27%) | 58 (22.22%) | 0.909 |
| Mydriatic-Cycloplegic | | 1 (0.0%) | 0 (0.0%) | 1 (0.4%) | 0 (0.0%) | 0.492 |
| Myorelaxant | | 5 (1.0%) | 2 (0.66%) | 1 (0.4%) | 2 (0.77%) | 0.909 |
| Mucolytic | | 3 (0.0%) | 0 (0.0%) | 2 (0.81%) | 1 (0.38%) | 0.492 |
| Parenteral nutrition | | 1 (0.0%) | 1 (0.33%) | 0 (0.0%) | 0 (0.0%) | 0.561 |
| Supplemental Oxygen | | 7 (1.0%) | 4 (1.33%) | 2 (0.81%) | 1 (0.38%) | 0.596 |
| Thyroid hormones | | 72 (9.0%) | 24 (7.97%) | 21 (8.5%) | 27 (10.34%) | 0.711 |
| Anti-Growth Hormone | | 1 (0.0%) | 0 (0.0%) | 1 (0.4%) | 0 (0.0%) | 0.492 |
| Bisphosphonates | | 12 (1.0%) | 1 (0.33%) | 6 (2.43%) | 5 (1.92%) | 0.286 |
| Prokinetic | | 2 (0.0%) | 1 (0.33%) | 1 (0.4%) | 0 (0.0%) | 0.711 |
| Psychostimulants | | 2 (0.0%) | 1 (0.33%) | 1 (0.4%) | 0 (0.0%) | 0.711 |
| Bacterial vaccines | | 1 (0.0%) | 0 (0.0%) | 0 (0.0%) | 1 (0.38%) | 0.492 |
| Vitamins | | 38 (5.0%) | 11 (3.65%) | 10 (4.05%) | 17 (6.51%) | 0.492 |
| Number of anticoagulants or antiplatelets | | 0.64 (± 0.56) | 0.74 (± 0.55) | 0.6 (± 0.54) | 0.55 (± 0.57) | **0.001*** |
| N° of drugs | | 3.47 (± 1.98) | 3.78 (± 2.01) | 3.35 (± 2.03) | 3.23 (± 1.85) | **0.014*** |
| **Topography** | | | | | | |
| Frontal | | 226 (28.0%) | 121 (40.2%) | 62 (25.1%) | 43 (16.48%) | **< 0.001*** |
| Temporal | | 223 (28.0%) | 113 (37.54%) | 67 (27.13%) | 43 (16.48%) | **< 0.001*** |
| Parietal | | 329 (41.0%) | 136 (45.18%) | 94 (38.06%) | 99 (37.93%) | 0.343 |
| Occipital | | 123 (15.0%) | 45 (14.95%) | 32 (12.96%) | 46 (17.62%) | 0.492 |
| Brainstem | | 29 (4.0%) | 17 (5.65%) | 11 (4.45%) | 1 (0.38%) | **0.016*** |
| Basal Ganglia | | 279 (34.0%) | 107 (35.55%) | 81 (32.79%) | 91 (34.87%) | 0.864 |
| Cerebellum | | 70 (9.0%) | 14 (4.65%) | 18 (7.29%) | 38 (14.56%) | **0.001*** |
| Side | Bilateral | 95 (11.74%) | 25 (8.31%) | 39 (14.94%) | 31 (12.55%) | 0.165 |
|  | Left | 387 (47.84%) | 137 (45.51%) | 126 (48.28%) | 124 (50.2%) |  |
|  | Right | 327 (40.42%) | 139 (46.18%) | 96 (36.78%) | 92 (37.25%) |  |

Characteristics of the 809 patients consecutively admitted in medical or surgical wards for the management of a spontaneous intracerebral hematoma (ICH). Categorical and continuous variables are respectively reported as number of patients (%) and mean (± SD). * Significant at p ≤ 0.05 after Holm-Bonferroni correction. **IVH**: intraventricular hemorrhage; **SAH**: subarachnoid hemorrhage; **GCS**: Glasgow Coma Scale; **TIA**: transient ischemic attack; **ENT**: ear, nose, and throat; **BPH**: benign prostatic hyperplasia; **NSAID**: nonsteroidal anti-inflammatory drugs; **DVT:** Deep venous thrombosis.
