# Supplementary material for: Machine learning model prediction of 6-month functional outcome in elderly patients with intracerebral hemorrhage
Source: Neurosurg Rev. 2022 May 6;45(4):2857–67. doi: 10.1007/s10143-022-01802-7 (PMC9349060; doi:10.1007/s10143-022-01802-7)
Supplement: Supplementary file 2 — Supplementary file2 (DOCX 19 KB) [file 10143_2022_1802_MOESM2_ESM.docx]

**Supplementary Table 2.** Multivariate logistic regression analysis.

| **Outcome** | **Parameter** | | **Odds Ratio** | **95% CI** | **p-value** |
| --- | --- | --- | --- | --- | --- |
| **Death** | Antiplatelet | | 1.127 | 0.560 - 2.270 | 0.737 |
|  | Renal Insufficiency | | 1.983 | 0.639 - 6.158 | 0.236 |
|  | Neurological | | 0.777 | 0.407 - 1.485 | 0.446 |
|  | Anticoagulant/Antiplatelet | | 1.67 | 0.784 - 3.557 | 0.183 |
|  | Antacids | | 1.475 | 0.778 - 2.798 | 0.234 |
|  | Frontal | | 1.226 | 0.652 - 2.306 | 0.528 |
|  | Temporal | | 1.195 | 0.650 - 2.195 | 0.566 |
|  | Brainstem | | 8.173 | 0.833 - 80.189 | 0.071 |
|  | Cerebellum | | 0.380 | 0.143 – 0.999 | **0.049** |
|  | IVH | | 1.38 | 0.692 - 2.753 | 0.360 |
|  | SAH | | 1.338 | 0.592 - 3.026 | 0.484 |
|  | Age | | 1.05 | 1.004 - 1.097 | **0.033** |
|  | Number of anticoagulants or antiplatelets | | 0.784 | 0.351 - 1.747 | 0.551 |
|  | Charlson Comorbidity Index | | 1.088 | 0.962 - 1.230 | 0.179 |
|  | N° Comorbidities | | 0.952 | 0.778 - 1.166 | 0.637 |
|  | N° of drugs | | 1.108 | 0.963 - 1.274 | 0.151 |
|  | Hematoma volume (cm^3^) | | 1.020 | 1.008 - 1.033 | **0.001** |
|  | GCS at admission | | 0.651 | 0.574 - 0.737 | **< 0.001** |
|  | ICH score (0 - 5 pt.) | | 1.673 | 1.187 - 2.358 | **0.003** |
|  | Pupillary status at admission | Isochoric | 1 | - | - |
|  |  | Anisocoric | 1.934 | 0.585 - 6.396 | 0.280 |
|  |  | Mydriatic | 1.872 | 0.178 - 19.678 | 0.601 |
|  |  | Miotic | 1.438 | 0.269 - 7.681 | 0.671 |
| **Poor outcome** | Antiplatelets | | 1.266 | 0.699 - 2.294 | 0.436 |
|  | Renal Insufficiency | | 2.203 | 0.870 - 5.573 | 0.095 |
|  | Neurological | | 1.315 | 0.797 - 2.169 | 0.284 |
|  | Anticoagulant/Antiplatelet | | 1.186 | 0.628 - 2.239 | 0.599 |
|  | Antacids | | 1.027 | 0.590 - 1.79 | 0.924 |
|  | Frontal | | 1.255 | 0.736 - 2.141 | 0.404 |
|  | Temporal | | 1.315 | 0.781 - 2.213 | 0.303 |
|  | Brainstem | | 10.834 | 1.278 - 91.851 | **0.029** |
|  | Cerebellum | | 0.62 | 0.304 - 1.262 | 0.187 |
|  | IVH | | 1.384 | 0.744 - 2.575 | 0.305 |
|  | SAH | | 1.667 | 0.833 - 3.339 | 0.149 |
|  | Age | | 1.057 | 1.017 - 1.098 | **0.005** |
|  | Number of anticoagulants or antiplatelets | | 0.661 | 0.329 - 1.329 | 0.245 |
|  | Charlson Comorbidity Index | | 1.211 | 1.096 - 1.337 | **<0.001** |
|  | N° Comorbidities | | 1.099 | 0.938 - 1.289 | 0.243 |
|  | N° of drugs | | 0.968 | 0.860 - 1.088 | 0.583 |
|  | Hematoma volume (cm^3^) | | 1.012 | 1.001 - 1.024 | **0.036** |
|  | GCS at admission | | 0.760 | 0.677 - 0.855 | **<0.001** |
|  | ICH score (0 - 5 pt.) | | 1.180 | 0.864 - 1.613 | 0.299 |
|  | Pupillary status at admission | Isochoric | 1 | - | - |
|  |  | Anisocoric | 0.771 | 0.233 - 2.557 | 0.671 |
|  |  | Mydriatic | 0.641 | 0.056 - 7.385 | 0.721 |
|  |  | Miotic | 0.562 | 0.100- 3.150 | 0.513 |

All parameters showing a statistically significant association (corrected p ≤ 0.05) at univariate analysis were included in the multivariate logistic regression analysis. **IVH**: intraventricular hemorrhage; **SAH**: subarachnoid hemorrhage; **GCS**: Glasgow Coma Scale; **ICH**: intracerebral hematoma.
